# Comparison of Leaf and Fine Root Traits Between Annuals and Perennials, Implicating the Mechanism of Species Changes in Desertified Grasslands

**DOI:** 10.3389/fpls.2021.778547

**Published:** 2022-02-04

**Authors:** Zhiying Ning, Yulin Li, Xueyong Zhao, Dan Han, Jin Zhan

**Affiliations:** ^1^Naiman Desertification Research Station, Northwest Institute of Eco-Environment and Resources, Chinese Academy of Sciences, Lanzhou, China; ^2^University of Chinese Academy of Sciences, Beijing, China; ^3^Yangling Agricultural Hi-Tech Industries Demonstration Zone, Xi’an, China

**Keywords:** leaf, fine root, plant functional traits, annuals, perennials, species changes, arid and semi-arid area

## Abstract

Annual species show traits, such as shortleaf lifetimes, higher specific leaf area, and leaf nutrient concentrations, that provided a more rapid resource acquisition compared to perennials. However, the comparison of root traits between the annuals and perennials is extremely limited, as well as the trade-offs of leaf and fine root traits, and resource allocation between leaf and root, which may provide insight into the mechanism of species changes in arid and semi-arid areas. With lab analysis and field observation, 12 traits of leaf and fine root of 54 dominant species from Horqin Sandy Land, Northeastern China were measured. The organization of leaf and fine root traits, and coordination between leaf and fine root traits of annual and perennial plants were examined. Results showed that there were differences between annuals and perennials in several leaves and fine root traits important in resource acquisition and conservation. Annuals had higher leaf area (LA), specific LA (SLA), and specific root length (SRL) but lower leaf dry-matter content (LDMC), leaf tissue density (LTD), leaf carbon concentration (LC), and fine root dry-matter content (FRDMC) than perennials. Leaf nitrogen (LN) concentration and fine root nitrogen concentration (FRN) were negatively related to LTD and FRDMC in annuals, while FRN was positively related to FRTD and fine root carbon concentration (FRC), and LA was positively related to LN in perennials. These implied that annuals exhibited tough tissue and low palatability, but perennials tend to have smaller leaves to reduce metabolism when N is insufficient. Annuals showed significant positive correlations between FRC/FRDMC and LDMC/LTD/LC, suggesting a proportional allocation of photosynthate between leaf and fine root. In perennials, significant negative correlations were detected between LN, LC, and SRL, fine root tissue density (FRTD), as well as between LA and FRTD/FRC. These indicated that perennials tend to allocate more photosynthate to construct a deeper and rigid roots system to improve resource absorption capacity in resource-limited habitats. Our findings suggested that annuals and perennials differed considerably in terms of adaptation, resource acquisition, and allocation strategies, which might be partly responsible for species changes in desertified grasslands. More broadly, this work might be conducive to understand the mechanism of species changes and could also provide support to the management and restoration of desertified grassland in arid and semi-arid areas.

## Introduction

Species changes are universal in degraded ecosystems, especially in desertified grassland in arid and semi-arid areas (Kerley and Whitford, 2009; Pfeiffer et al., 2019). Dominant species in grassland changed from perennial grasses to annual forbs and grasses after grazing in western Oklahoma, United States (Collins et al., 1988). Herbaceous species, especially grasses, were lost and replaced by xerophytic shrubs or semi-shrubs during the degradation development in the cold semi-arid grasslands of Qinghai-Tibet Plateau, North-west China (Li et al., 2006). Understanding the mechanisms underlying these changes has been a challenge for ecologists. Previous studies suggested that the species changes during grassland desertification are the results of the interaction between species adaptation and environmental changes (D’Odorico et al., 2013; Zhang et al., 2020). Plant growth is often limited by drought stress and nutrient deficiency in arid and semi-arid areas, which have been exacerbated due to grassland desertification (Bennett and Adams, 2001; Yair and Kossovsky, 2002; Dong et al., 2009). Generally, functional traits have been used as indicators of plant species response to environmental change for characterizing plant survival and resource use strategies under stress conditions (Dyer et al., 2001; Reich et al., 2003). Previous studies compared photosynthetic, leaf morphology, and chemistry traits in annuals and perennials (Jaikumar et al., 2013; González-Paleo and Ravetta, 2018; González-Paleo et al., 2019). Nonetheless, few have specialized in the differences in coordination and organization in leaf and fine root traits between annuals and perennials, and very few have linked these with species changes. This study explores the plant adaptive mechanisms and species assembly rules by addressing the question as to whether there are differences in trade-offs of leaf and fine root traits between annuals and perennials.

Abundant pieces of evidence suggested that for plants, annual and perennial life histories exhibit leaf traits trade-offs between the rapid acquisition of resources and conservation of resources (Diaz et al., 2004; Wright et al., 2004a; Monroe et al., 2019). Annuals with shortleaf lifetimes, high SLA and leaf N concentrations, and fast returns to light and CO_2_ generally capture resources and complete their life cycles more rapidly (Wright et al., 2004b), while perennials exhibit robustness and low palatability allow continued leaf function to maximize resource conservation and have adaptive advantages in resource-limited conditions (González-Paleo et al., 2019). However, compared to leaf traits, fewer studies have focused on traits of underground organs of annuals and perennials (Roumet et al., 2006; DuPont et al., 2014). It is not clear whether traits of root exhibit similar trade-offs (between below-ground resource acquisition and resource conservation, and root persistence) to leaf. Yet, there is some evidence suggesting that suites of correlated root traits are linked to plant growth strategies. Fort et al. (2012) found that drought-sensitive species have higher SRL, revealing more opportunistic root strategies than drought-tolerant species. Laughlin et al. (2021) demonstrated that forest species in warm climates tend to have low SRL and high root tissue density (RTD), but species in cold climates exhibit a high SRL and low RTD. In addition, roots directly access underground resources (nutrients and water) and hence better reflect plant underground components in response to the environmental changes and the adaptive strategy of plants to stressed conditions in desertified grassland (Hodge, 2004; Bardgett et al., 2014). The most obvious changes during grassland desertification are the decrease in productivity and selective loss of nutrient-rich fine soil particles which lead to seriously coarse-textured soil, poor soil nutrients, and soil water retention (McLendon and Redente, 1992; Zuo et al., 2009). Plant growth is mainly limited by drought stress and nutrient deficiency rather than light and CO_2_ in desertified grassland in arid and semi-arid grasslands. We, therefore, tested whether fine root traits and organization of fine root traits differ between annuals and perennials, which might give insights in understanding species assembly in arid and semi-arid areas.

If root traits between annuals and perennials are subjected to the same trade-offs as leaf traits (resource acquisition and resource conservation), differences in leaf-root traits coordination between annuals and perennials are expected, with leaf-root traits exhibiting extravagant resource allocation strategy between above- and belowground components in annuals, and resource conservation allocation strategy in perennials. Growing evidence of consistent trait syndromes between leaf and root indicates coordination between above- and belowground organs in terms of the acquisition and allocation of limited resources (Liu et al., 2010; de la Riva et al., 2016). However, there are more reasons to expect that trade-offs between leaf and fine root traits of annual species might differ considerably from those of perennials. Literature demonstrated that this coordination varied between growth forms and strategies. For example, Valverde-Barrantes et al. (2021) found that among woody plants, high LN is associated with low RTD, whereas among non-woody plants, low SRL is associated with high LN. Fort et al. (2012) showed that for the same SLA, drought-tolerant species exhibit higher root mass and lower SRL than drought-sensitive species, to develop and maintain a coarser and deeper root system. These trade-offs are the result of plant species adapting to resource-poor habitats by improving their resource-acquisition ability. In addition, trade-offs between leaf and root traits might be also altered due to changes in the environment owing to climatic or edaphic shifts. Results from Liu et al. (2010) suggested that the ratio of leaf N per area root N per length increased, but the ratios of SLA to SRL and leaf N to root N decreased from semi-arid to arid environment. These changes are associated with the allocation of photosynthates and N to root, likely reflecting that those plants tend to allocate more resources to the construction of root systems due to extremely dry conditions (Craine, 2006; Morales et al., 2015). We surmised that different patterns of resource allocation between leaf and root might be observed in annuals and perennials, in response to stressed habitat, which might be conducive to further understanding of adaptation strategies of annual and perennial species in desertified grassland.

This study investigated 12 traits of leaf and fine root of 54 dominant species in Horqin Sandy Land, Northeastern China, to analyze leaf and fine root traits organization and leaf-root traits coordination in annuals and perennials, respectively. Horqin Sandy Land is one of the four well-known Sandy Lands in northern China, has been subjected to serious desertification in the last 40 years (Duan et al., 2014). Studies in this area have found that the dominance of annual species decreased but that of perennial species increased with the desertification development (Zhang et al., 2004; Zuo et al., 2009; Ning et al., 2021). These might be attributed to the adaptation of plant species to environmental changes, as well as different resource use strategies. Our objective is to explore plant strategies of resource use and allocation in annuals and perennials, by using a functional approach, thereby implicating the mechanism of species changes in desertified grassland. Specifically, we hypothesize that: (a) Annual species differ from perennial species in fine root traits organization in desertified grassland in arid and semi-arid areas; (b) The coordination between leaf and fine root traits of the annuals and perennials differs in ways that may reflect different resource allocation strategies.

## Materials and Methods

### Study Area

The study was conducted in the Horqin Sandy Land (118.4°E to 123.5°E, 42.7°N to 45.8°N, 180 to 650 masl), which covers an area of 12.90 × 10^4^ km^2^ in the northeastern part of Inner Mongolia, China. The area has a continental semi-arid to a semi-humid monsoon climate. The mean annual temperature ranges from 5.8°C to 6.4°C, with mean monthly temperatures from −12.6°C in January to 23.5°C in August, and mean annual precipitation from 343 mm to 451 mm, of which approximately 70% falls from June to August. The mean annual potential evaporation ranges from 1,500 to 2,500 mm. The mean annual wind velocity ranges from 3.5 to 4.5 m s^–1^, and the mean wind velocity in spring (the season with the lowest vegetation cover and thus, the greatest vulnerability to erosion) ranges from 4.2 to 5.9 m s^–1^. Windy days with a velocity greater than 17 m s^–1^ is from 25 to 40 days per year, which results in 10 to 15 days of sandstorms and dust storms, mainly (more than 70% of the total) in the spring.

The Horqin Sandy Land is a sandy dune landscape due to desertification, with a mosaic distribution of flat or undulating sandy land, mobile dunes, semi-fixed dunes, fixed dunes, and interdune lowlands. The soil in the study area is classified as a Cambic Arenosol (FAO and ISRIC, 1988), in which coarse sand with a particle size of 0.25 to 1 mm accounts for 20 to 58% of the mass, fine sand with a particle size of 0.05 to 0.25 mm accounts for 40 to 67%, and clay and silt with a particle size <0.05 mm accounts for 0.1 to 15%. The soil organic matter content is 0.08 to 0.49%. The dominant species in this area are *Agriophyllum squarrosum* (annual forb), *Setaria viridis* (annual grass), *Artemisia halodendron* (sub-shrub), *Caragana microphylla* (shrub), *Bassia dasyphylla* (annual forb), *Artemisia annua* (annual forb), *Chenopodium acuminatum* (annual forb), *Artemisia frigid* (annual forb), *Periploca sepium* (shrub), and *Cynanchum thesioides* (annual forb).

### Leaf and Fine Root Traits Measurement

In August 2018, we investigated the traits of leaf and fine root of 54 dominant plants on a sand streak in Horqin Sandy Land. We measured leaf and fine root traits on five samples for each species, and at least five healthy and mature individuals were collected as one sample. To obtain intact fine roots, we excavated each plant by digging soil range 25 cm diameter and 25 cm deep, and wider and deeper when necessary to obtain fine roots for deeper-rooted species or taprooted species. For some sand-fixing shrubs, such as *A. halodendron* and *C. microphylla*, it was necessary to excavate to a depth of 1 m to obtain complete fine root tissue. Plants were transported to the laboratory and cleaned with distilled water to remove adherent soil, debris, and root of surrounding plants. For leaves, intact and mature leaves were collected for each sample. Part of the collection was oven-dried at 65°C for chemical analysis, while the other (10 leaves, or more for microphyllous species) was placed in a plastic bag for traits analysis. For fine root, we extracted undamaged fine root (<2 mm) of each sample. Part of the collection was oven-dried at 65°C for chemical analysis, while the other was placed in a plastic bag for traits analysis. Then, the collection of leaves and fine roots of each sample were put in the water in the dark for 24 h at 4°C to allow water saturation. For the leafless species *Ephedra sinica*, the plant part functionally similar to the leaf was measured.

Before measurement, fresh and water-saturated leaf and fine root were dried with clean filter paper gently and weighted for water-saturated leaf and fine root. The leaf and fine root of each sample were then scanned with a scanner (Epson, Japan) for the digital image. Image analysis was conducted with WinRHIZO software (Regent Instruments, Quebec, Canada) to measure the LA, length, and volume of the fine root. After scanning, these materials were oven-dried at 65°C to a constant weight and weighted. Leaf thickness (LT) was measured with a digital caliper as the mean of 3 measurements for each leaf, and 10 leaves were measured for each sample. SLA and SRL were calculated as the ratio between leaf area and leaf dry mass, and between root length and root dry mass, respectively. LDMC and FRDMC were calculated as the ratio between leaf and fine root dry mass and their water-saturated mass, respectively. LTD and FRTD were calculated as the ratio between leaf dry mass and leaf volume, and between fine root dry mass and root volume, respectively. The leaf and fine root of each sample were ground into a fine powder using a ball mill (GT300 Ball Mill, POWTEQ, Beijing, China). The C and N concentrations of leaf and fine root (LC, LN, FRC, and FRN) were measured by an elemental analyzer (ECS4010, Costech, Milan, Italy).

### Calculations and Data Analysis

Phylogenetic signal (Blomberg’s *K*) in all leaf and fine root traits was tested using the *K* statistic, where *K* is calculated as the ratio of the observed phylogenetically correct mean-square error divided by the mean-square error of the data, standardized by the expectation under Brownian motion (Blomberg et al., 2003). When measuring functional traits with phylogenetic signals, the species in the phylogenetic tree are usually randomly permuted 999 times to calculate the *K* statistic. The higher the *K*-value, the stronger the phylogenetic signal is and suggested the tendency of close relatives to have similar traits due to their common ancestor. The *K*-value and associated *P*-value were calculated with 3.5.3 of R^1^, using the ‘picante’ package.

When the statistical analysis is conducted at the species level, traits of plant species are not statistically independent because species have a common ancestor and with different degrees of genetic relationship among species (Felsenstein, 1985). Thus, the phylogenetically independent contrasts (PIC) are essential to enhance the reliability of statistical analysis and reveal the potential importance of evolutional history in determining the current traits of species. A phylogenetic tree was recovered for 54 species by using the Phylo-matic tree^2^ (Webb and Donoghue, 2005), based on the phylogenetic hypothesis for relationships among angiosperm families. For this analysis, branch lengths were estimated from a set of dated nodes based on fossil calibrations of vascular plants (Webb et al., 2011). PIC of each trait was calculated as the difference in mean trait values for pairs of sister species and nodes. We calculated the PIC of leaf and fine root traits of 54 species using the ‘ape’ package in R.

Coefficients of variation (CV) in leaf and fine root traits were calculated using Equation 1:


(1) CV(%)=SD/mean×100

The 54 species in Horqin Sandy Land were classified into two categories: annuals (27) and perennials (27) (Supplementary Table A. 1). For all the variables measured, the distribution of values was tested for normality (Kolmogorov–Smirnov test, *P* = 0.05) and the homogeneity of error variance (using Levene’s test, *P* = 0.05). An independent-sample *t*-test was used to test for the differences in leaf and fine root traits between annuals and perennials. The Pearson correlation tests were used to analyze the correlations among leaf and fine root traits. We repeated this correlation analysis using PIC of traits to test the effect of species evolutional history in the correlation of leaf and fine root traits. To test the correlations both within and among traits defining leaf and fine root, a principal component analysis (PCA) was performed on 12 traits (with ‘vegan’ package in R). To account for the influence of species evolutionary histories, we conducted PCA both with and without the PIC. Both PIC correlations and PCA with PIC were analyzed based on standardized contrasts, calculating relationships through the origin and adjusting degrees of freedom.

## Results

### Variation of Leaf and Fine Root Traits

The LT and LA showed large variation (CV) among species, while FRDMC, LC, FRC, LN, and FRN had low variation in 54 dominant species in Horqin Sand Land (Table 1). Significant phylogenetic signals in LDMC, SLA, FRTD, and FRC were found (*P* < 0.05), suggesting that these traits were strongly influenced by their evolutionary history. However, the *K* values of these traits were relatively small (*K* < 1).

**TABLE 1:** Traits of leaf and fine root of 54 dominant species in Horqin Sandy Land and their phylogenetic signal (*K*).

Traits	Traits	Mean ± SE	Minimum	Maximum	*CV*(%)	*K*	*P*
Leaf thickness (mm)	LT	0.26 ± 0.03	0.09	1.45	92.31	0.24	0.19
Leaf area (cm^2^)	LA	6.69 ± 1.50	0.08	60.07	164.57	0.11	0.09
Leaf dry-matter content (g/g)	LDMC	0.24 ± 0.01	0.07	0.47	41.67	0.06	0.02
Specific leaf area (cm^2^/g)	SLA	219.74 ± 11.53	31.83	498.56	38.56	0.08	0.08
Leaf tissue density (g/cm^3^)	LTD	0.03 ± 0.00	0.01	0.06	33.33	0.06	0.02
Fine root dry-matter content (g/g)	FRDMC	0.34 ± 0.01	0.13	0.60	26.47	0.04	0.12
Specific root length (cm/g)	SRL	1258.20 ± 107.15	375.90	4147.92	62.58	0.03	0.46
Fine root tissue density (g/cm^3^)	FRTD	0.23 ± 0.01	0.09	0.55	39.13	0.08	0.01
Leaf nitrogen concentration (%)	LN	2.36 ± 0.09	0.75	3.87	28.39	0.02	0.59
Leaf carbon concentration (%)	LC	43.56 ± 0.47	35.63	49.89	7.85	0.05	0.13
Fine root nitrogen concentration (%)	FRN	1.03 ± 0.07	0.11	2.46	24.27	0.04	0.27
Fine root carbon concentration (%)	FRC	45.73 ± 0.24	41.65	50.76	3.94	0.06	0.04

*K, phylogenetic signal.*

**FIGURE 1:**
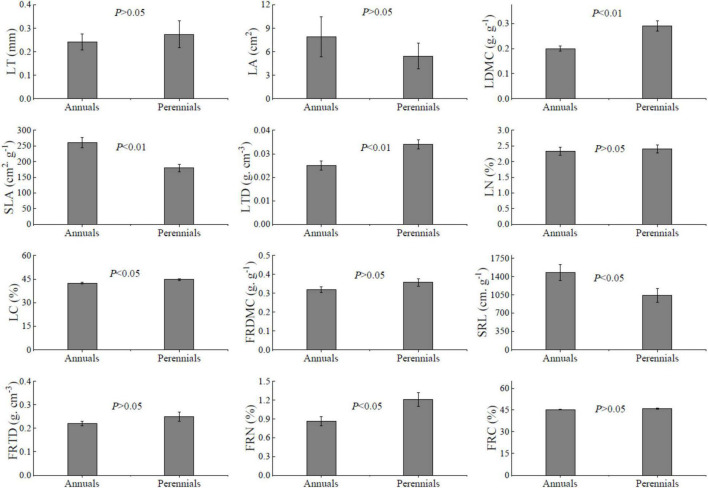
Traits of leaf and fine root of 54 dominant species. LT, leaf thickness; LA, leaf area; LDMC, leaf dry-matter content; SLA, specific leaf area; LTD, leaf tissue density; FRDMC, fine root dry-matter content; SRL, specific root length; FRTD, fine root tissue density; LN, leaf nitrogen concentration; LC, leaf carbon concentration; FRN, fine root nitrogen concentration; FRC, fine root carbon concentration. Values are mean ± SE. Independent-sample *t*-test, *P* < 0.05 represents a significant difference between annuals and perennials.

Annuals showed significantly higher SLA and SRL than perennials (Figure 1, *P* < 0.05). But the perennial species had higher LDMC, LTD, LC, FRN, and FRC than annual species significantly (*P* < 0.05). Unexpectedly, there was no significant LN difference between annuals and perennials, as well as LT, LA, FRDMC, and FRTD (*P* > 0.05).

### Correlations Among Leaf and Fine Root Traits

Pairwise correlation among leaf and fine root traits of overall species were analyzed, significant correlations were found (Table 2, *P* < 0.05). LDMC was positively related to LTD and LC but negatively related to SLA and LN (*P* < 0.05). The correlation of fine root traits had a similar pattern as the leaf. FRDMC was positively related to FRTD but negatively related to SRL (*P* < 0.05). When PIC was included in the analysis of pairwise correlation of leaf and fine root traits, the different patterns of correlation were observed (Table 2). The correlations between LDMC and SLA, between LTD and LC, LN, and between SLA and FRTD disappeared after considering evolutionary relationships among species. But the SLA, FRTD, and FRDMC were associated with FRN (*P* < 0.05).

**TABLE 2:** Matrix of Pearson’s correlation coefficients for the relationships between leaf and fine root traits of 54 dominant species (*n* = 54).

Trait	LT	LA	LDMC	SLA	LTD	LN	LC	FRDMC	SRL	FRTD	FRN	FRC
LT		−0.34*	−0.57**	–0.04	−0.45**	–0.18	−0.85**	0.36*	–0.14	0.00	–0.06	0.13
LA	–0.10		0.22	0.01	0.02	0.53**	0.29*	−0.44**	0.25	−0.48**	0.42**	−0.44**
LDMC	−0.31*	–0.23		–0.18	0.31*	0.40**	0.70**	–0.17	–0.15	–0.14	0.40**	−0.49**
SLA	−0.27*	–0.04	−0.43**		−0.68**	0.19	0.14	0.13	–0.03	–0.21	0.06	–0.09
LTD	−0.52**	–0.00	0.76**	−0.41**		–0.12	0.18	–0.24	0.22	0.35**	–0.14	0.17
LN	0.10	0.10	–0.14	0.10	−0.33*		0.34*	−0.52**	0.29*	−0.58**	0.80**	−0.66**
LC	−0.30*	–0.16	0.52**	–0.07	0.39**	0.18		–0.26	0.11	–0.12	0.25	−0.35**
FRDMC	−0.34*	−0.27*	0.64**	–0.10	0.51**	–0.19	0.28		–0.24	0.45**	−0.46**	0.23
SRL	–0.17	0.14	–0.04	0.17	0.20	−0.35**	−0.31*	0.06		–0.22	0.42**	–0.20
FRTD	0.04	–0.18	0.10	−0.28*	–0.02	0.32*	0.14	0.34*	−0.57**		−0.66**	0.48**
FRN	0.18	–0.00	0.08	–0.21	0.09	0.62**	0.22	–0.12	–0.26	0.31*		−0.78**
FRC	–0.14	−0.32*	0.35**	–0.15	0.23	0.10	0.42**	0.22	–0.25	0.19	0.18	

*P < 0.05; **P < 0.01. Below-diagonal values and above-diagonal values indicate correlations without and with phylogenetically independent contrasts, respectively. LT, leaf thickness; LA, leaf area; LDMC, leaf dry-matter content; SLA, specific leaf area; LTD, leaf tissue density; FRDMC, fine root dry-matter content; SRL, specific root length; FRTD, fine root tissue density; LN, leaf nitrogen concentration; LC, leaf carbon concentration; FRN, fine root nitrogen concentration; FRC, fine root carbon concentration.

Both for annuals and perennials, species with high LT tend to low LDMC and LTD, and species with high SRL means low FRTD (Tables 3, 4). However, annuals and perennials showed different patterns of correlation among leaf traits or fine root traits. In annuals, LN was negatively related to LTD, and FRN was negatively related to FRTD (Table 4, *P* < 0.05). But in perennials, LN was positively correlated with LA (Table 3, *P* < 0.05). And FRN is positively correlated with FRTD and FRC.

**TABLE 3:** Matrix of Pearson’s correlation coefficients for the relationships between leaf and fine root traits in perennials (*n* = 32).

Trait	LT	LA	LDMC	SLA	LTD	LN	LC	FRDMC	SRL	FRTD	FRN
LA	–0.05										
LDMC	–0.39	–0.12									
SLA	–0.34	–0.27	–0.27								
LTD	−0.62**	0.18	0.74**	–0.24							
LN	0.10	0.42*	–0.09	0.08	0.37						
LC	0.09	–0.39	0.48*	–0.18	0.19	0.35					
FRDMC	−0.43*	–0.38	0.35	0.11	0.40	–0.06	0.30				
SRL	–0.15	0.42*	–0.05	0.02	0.20	−0.51**	−0.47*	–0.06			
FRTD	0.15	−0.44*	0.18	–0.22	0.13	−0.42*	0.37	0.50**	−0.51**		
FRN	0.21	–0.22	0.05	–0.36	–0.13	0.70**	0.30	–0.07	–0.29	0.39*	
FRC	–0.09	−0.50**	0.16	–0.18	0.02	0.29	0.31	0.04	–0.38	0.25	0.39*

*P < 0.05; **P < 0.01. LT, leaf thickness; LA, leaf area; LDMC, leaf dry-matter content; SLA, specific leaf area; LTD, leaf tissue density; FRDMC, fine root dry-matter content; SRL, specific root length; FRTD, fine root tissue density; LN, leaf nitrogen concentration; LC, leaf carbon concentration; FRN, fine root nitrogen concentration; FRC, fine root carbon concentration.

**TABLE 4:** Matrix of Pearson’s correlation coefficients for the relationships between leaf and fine root traits in annuals (*n* = 22).

Trait	LT	LA	LDMC	SLA	LTD	LN	LC	FRDMC	SRL	FRTD	FRN
LA	–0.16										
LDMC	−0.41*	–0.28									
SLA	–0.24	–0.04	–0.26								
LTD	−0.63**	–0.04	0.74**	–0.33							
LN	0.10	0.30	–0.33	0.19	−0.38*						
LC	−0.70**	–0.01	0.42**	0.28	0.38*	0.04					
FRDMC	−0.42*	–0.18	0.66**	–0.10	0.51**	–0.33	0.09				
SRL	–0.19	0.11	0.27	0.06	0.32	–0.21	–0.09	0.39*			
FRTD	0.47*	–0.11	–0.23	–0.28	–0.34	0.18	–0.20	–0.04	−0.66**		
FRN	0.07	0.31	–0.38	0.33	–0.33	0.60**	–0.08	−0.49**	–0.04	–0.00	
FRC	–0.31	–0.37	0.48*	0.09	0.42*	–0.20	0.48*	0.40*	0.00	–0.03	−0.49**

*P < 0.05; **P < 0.01. LT, leaf thickness; LA, leaf area; LDMC, leaf dry-matter content; SLA, specific leaf area; LTD, leaf tissue density; FRDMC, fine root dry-matter content; SRL, specific root length; FRTD, fine root tissue density; LN, leaf nitrogen concentration; LC, leaf carbon concentration; FRN, fine root nitrogen concentration; FRC, fine root carbon concentration.

### Coordination Between Leaf and Fine Root Traits

Looking at the relationship between leaf and fine root traits for overall species, there was a positive correlation between LDMC and FRDMC, as well as between LN and FRN, and between LC and FRC (Table 2, *P* < 0.05). However, SLA and LTD did not correlate to SRL and FRTD, respectively (*P* > 0.05). When PIC was included, the SLA and SRL were still not correlated, while the relationship between LN and FRN exhibited concordant patterns as ahistorical correlations, although the magnitude was increased. The LTD was positively correlated with FRTD, but the LDMC was decoupled from FRDMC. Somewhat unexpected was the positive relationship between LC and FRC in ahistorical correlations, while the negative relationship was observed in evolutionary correlations (Table 2).

The coordination between leaf and fine root traits of annuals differed from those of perennials. For annuals, LT was negatively related to FRDMC and FRC (Table 4, *P* < 0.05). And the LDMC and LTD were related to FRDMC and FRC positively (*P* < 0.05). The LN and LC were related to FRN and FRC, respectively, suggesting the proportional distribution of photosynthate and nutrients between the aboveground and belowground components in annuals. But for perennials, the LN was positively related to FRN, but negatively related to SRL and FRTD (Table 3, *P* < 0.05). A positive relationship between LA and SRL and a negative relationship between LA and FRTD and FRC were also detected in perennials (*P* < 0.05).

### Principal Component Analysis of Leaf and Fine Root Traits

**FIGURE 2:**
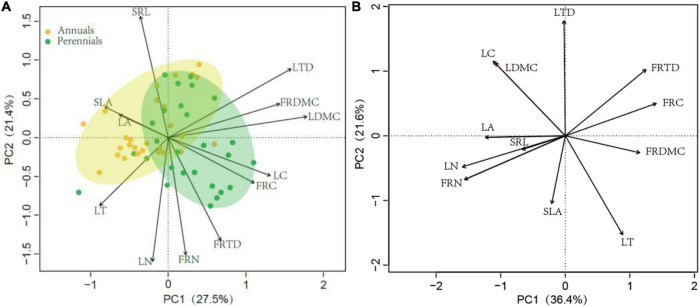
Principal component analysis (PCA) on traits in 54 dominant species in Horqin Sandy Land. Data: **(A)** without and **(B)** with phylogenetically independent contrasts. LT, leaf thickness; LA, leaf area; LDMC, leaf dry-matter content; SLA, specific leaf area; LTD, leaf tissue density; FRDMC, fine root dry-matter content; SRL, specific root length; FRTD, fine root tissue density; LN, leaf nitrogen concentration; LC, leaf carbon concentration; FRN, fine root nitrogen concentration; FRC, fine root carbon concentration.

The PCA of trait correlations was performed in leaf and fine root traits in 54 dominant species (Figures 2A,B). The first two axes of the PCA performed with leaf and fine root traits accounted for 50% of the variance. The first PCA axis (PC1) was a structural axis defined by LDMC, LTD, LC, and FRDMC; the second PCA axis (PC2) was defined by SRL and LN, FRN, and FRTD, but the SRL and other traits (LN, FRN, and FRTD) were in an opposite direction (Supplementary Table A. 3). Although there was a little overlap, traits of annuals and perennials were clustered and separated from each other. The annuals were mostly distributed on the left half of the PCA axis 2, while the perennials were mainly distributed on the right half of the PCA axis 2. Perennials had higher LTD, LDMC, LC, FRDMC, FRTD, and FRC than perennials. When including PIC in the PCA, the patterns of correlations among leaf and fine root traits were completely inconsistent, confirming that species evolutionary history influenced the observed correlations among leaf and fine root traits. The first and second PCA axis explained 58% of the total variation of all 12 leaf and fine root traits (Figure 2A). The LN, FRN, LA, and FRTD were loaded on the PCA axis 1, while the LT, LDMC, and LC were loaded on the PCA axis 2 (Supplementary Table A. 3).

## Discussion

### Annuals Differed From Perennials in Leaf and Fine Root Traits Important in Resource Uptake and Conservation

Differences in leaf and fine root traits between annuals and perennials followed the pattern of the gradient (from acquisitive to conservative) in carbon-use strategies previously reported (Wright et al., 2004b; González-Paleo and Ravetta, 2018). As expected, the annuals exhibited higher LA, SLA, and SRL but showed lower LDMC, LTD, and LC and FRDMC than perennials (Figure 1). These results suggested that annuals have traits that demonstrated a more acquisitive strategy than perennials since higher SLA and SRL have usually been associated with large leaf area and leaf gas exchange, rapid rates of root elongation, and high resource uptake capacities (Hodge, 2004; Roumet et al., 2006). By contrast, perennials leaf and fine root traits were characterized by high LDMC, LTD, LC, and FRDMC, which are associated with longer-lived leaf and root (Ryser, 1996; Tjoelker et al., 2005). Theoretically, the annuals have higher LN and FRN than perennials, implying that a higher proportion of the energy allocated to leaf and root construction is used for ‘expensive’ proteins for rapid exploitation and assimilation of resources (Eissenstat and Yanai, 1997; Martínez et al., 2002). However, there was no significant difference in LN between annuals and perennials. Perennials had a higher FRN than annuals unexpectedly (Figure 1). This might be due to the fact that 7 of the 22 perennials we investigated in this area were legumes, which are characterized by biological N fixation.

High LA, SLA, and SRL might have been selected to maximize leaf and root surface area in annuals, enabling a greater acquisition of carbon and resource which is crucial to grow fast and to complete their life cycle in a short period of time (Hodge, 2004; Roumet et al., 2006). However, high N and the combination of high SLA may increase leaf vulnerability to herbivory and physical hazards in annuals. By contrast, in perennials, the diversion of photosynthate promotes persistence, tolerates herbivory, and resists environmental stress *via* higher tissue density and dry matter content, which is the advantage of survival in harsh conditions of desertified grassland (González-Paleo et al., 2016). And perennials with higher FRDMC may be advantageous in terms of more efficient soil exploration and the ability to better penetrate the soil matrix, reflecting an adaptation to survive and water stress tolerance in environments where the competition is strong (Beyer et al., 2013; Liu et al., 2019). Meanwhile, higher FRDMC in perennials is associated with the longer-lived roots, which means a greater carbon storage capacity, and enough total carbon available for the next vegetation season (Munné-Bosch, 2014). PCA analysis also showed considerable differences in leaf and fine root traits between annuals and perennials (Figure 2), suggesting that annuals and perennials have different adaptation and resource acquisition strategies.

### The Annual Species Differed From Perennial Species in Leaf and Fine Root Traits Organization in Arid and Semi-Arid Grasslands

Surprisingly, significant relationships existed neither between SLA and LN nor between SRL and FRN in arid and semi-arid desertification in our study. The observed unwonted patterns of leaf and fine root traits in our study might be attributed to two reasons. First, functional trade-offs of traits and resource acquisition strategies were differed among functional groups due to their ecological specialization (Supplementary Table A. 2). For instance, the grasses (annual grasses and perennial grasses) had the highest SLA and SRL but the lowest LN and FRN, while the perennial forbs had the highest LN, FRN, and FRTD but the lowest SLA and SRL. The coexistence in desertified grassland of species with a multifaceted array of resource acquisition strategies was the key determinant of maintaining a range of niche conditions and system stability (Kong et al., 2014; Diaz et al., 2015; Kraft et al., 2015). Second, our principal component analysis failed to identify LN and FRN (Figure 2A), which support enzyme functioning and economy of resource capture in plant tissues, as significant contributors to the primary axis of leaf and fine root traits variation (Valverde-Barrantes et al., 2017). Instead, leaf and fine root traits were most significantly explained by structural traits. However, these were significantly explained by FRN and LN after considering PIC (Figure 2B), probably implying that the evolutionary history overshadowed the central role of nitrogen in metabolic activity in desertified grassland.

Negative correlations between FRTD and SRL, as well as between LT and LTD, and LC were observed both in annuals and perennials (Tables 3, 4). A general tendency for species inhabiting arid and semi-arid areas to have thick leaf blades, leathery leaves have been reported, which is beneficial for plants to withstand drought by reducing water loss, excessive irradiance, and heat load, and facilitating retention of water (Sobrado and Medina, 1980; Niinemets, 2001; Wright et al., 2001). And species with higher SRL in dry habitats are able to avoid drought by accessing deep water, as confirmed by their deep root system (Morales et al., 2015). Yet, it appears that annuals did not follow similar patterns to perennials when we explored the organization of leaf and fine root traits of annuals and perennials. In annuals, LN and FRN were negatively correlated with LTD and FRDMC, respectively (Table 4), probably due to the fact that species with high protein content (typically high-SLA leaf and/or high-SRL root) tend to have lower concentrations of other ‘expensive’ compounds, such as lignin, phenols, and lipids (Martínez et al., 2002). Perennials, however, showed a positive correlation between FRN and FRTD/FRC. A possible explanation for this unexpected result is that 7 of the 22 perennials we investigated were legumes (such as *Caragana microphylla*, *Lespedeza bicolor*), which capture additional N through biological N fixation. These legumes would have a growth advantage in N limited desertified grassland, which is theoretically one of the reasons why these species could colonize in desertified grassland. Furthermore, a positive correlation between LA and LN was observed in perennials. Leaf nitrogen is integral to protein factors in photosynthetic machinery, especially Rubisco (Wright et al., 2004a). This implied that as a natural basis for light interception and substance exchange of plants, leaves tend to be smaller to reduce metabolism, such as photosynthesis, when N is insufficient. And small leaf areas may cause a reduction of transpiration to protect against water loss, which is probably the plant’s adaptation to water scarcity in our study area (Bacelar et al., 2004). This trade-off of leaf traits might be contributed by adapting perennials in a resource-poor environment in arid and semi-arid areas.

### The Coordination Between Leaf and Fine Root Traits of Annuals and Perennials Differed in Ways in an Arid and Semi-Arid Grassland

Studies have examined the coordination between leaf and root traits for characterizing resource economic trade-offs and allocation, such as SLA and SRL, leaf and root nutrients concentrations (e.g., Tjoelker et al., 2005; Hajek et al., 2013). Contrary to previous studies (Withington et al., 2006; Geng et al., 2014), the SLA-SRL relation was not significant in our study regardless of whether the PIC was considered, even though we investigated 54 dominant species in our study area. The missing SLA-SRL relationship might be due to the inconsistent effects of environmental and evolutionary pressures on the plant above- and belowground components (Liu et al., 2010). LN was inversely correlated with FRN, and the correlation coefficient has increased by 90% after considering PIC, demonstrating that the nutrient allocation between leaf and fine root was the outcome of plant specialization toward soil fertility, instead of species common lineages. And no significant phylogenetic signal was detected in traits related to resource acquisition (SRL, SLA, FRN, and LN), probably implying that these traits were less affected by the degree of phylogenetic relatedness, and their phenotypic plasticity obscure phylogenetic signal. We also tested for coordination of structural traits between leaf and fine root. The LDMC was decoupled from FRDMC, the LTD was positively correlated with FRTD, and the LC-FRC correlation shifted from positive to negative when including PIC. Meanwhile, a significant phylogenetic signal was detected in structural traits (LDMC, LTD, FRTD, and FRC), suggesting the conservative evolution of these traits in desertified grassland, despite the presence of environmental variation and resource limitation. Ma et al. (2018) found that the variation in morphological traits (root diameter and SRL) was strongly influenced by evolutionary history at the global scale, while chemical traits did not show a significant phylogenetic signal. These results indicated that plants may show differentiation in plant traits depending on environmental conditions. Pfennig et al. (2006) proposed that divergence between species arising from trait plasticity could contribute to species coexistence. In desertified grassland, the differentiation in plant traits was occurred in resource-limited conditions, as a result of the ability of plasticity and adaptation to the environment.

When we explored the coordination between leaf and fine root traits of annuals and perennials, respectively, it appears that annuals did not follow similar patterns to perennials. Annuals showed significant positive correlations between FRC, FRDMC and LDMC, LTD and LC, suggesting a proportional allocation of photosynthate between leaf and fine root in annuals. However, a significant negative correlation was detected between LN, LC, and SRL, FRTD in perennials, as well as between LA and FRTD/FRC. These results implied a conservative resource allocation strategy that more photosynthate has been allocated to roots when leaf N is insufficient in desertified grassland, to maximize root length and resource capture and decrease potential growth rates. And perennials tend to have small leaves, to allocate more photosynthate to root establishment. Likewise, Fort et al. (2012) proposed that drought-tolerant species may allocate more biomass to root and invest more in vessel clarification to ensure water acquisition and transport in dry conditions. Drenovsky et al. (2008) have found that invasive perennial forbs allocated more biomass to roots and allocated proportionately more root length to nutrient-rich microsites than did natives, which might contribute to the success of invasive forbs in low-nutrient environments. These pieces of evidence altogether suggested that a deeper and rigid root system and small leaves for the same level of investment for unit mass in perennials might be produced and maintained, as well as a conservative strategy and competitive advantage in desertified grassland.

### Implications for Species Changes in Desertified Grassland in Arid and Semi-Arid Areas

In Horqin Sandy Land, a large amount of clay and silt particles have been removed by strong wind erosion resulting in coarse-textured soil, which is conducive to the infiltration of precipitation and replenishing of water into the deep soil layer (Cheng et al., 2020). The precipitation regimes that characterize arid and semi-arid grasslands are largely composed of small-sized precipitation events (<10 mm), which effectively improved shallow soil moisture, but only be accounted for a small proportion of the total rainfall (17%). The large-sized precipitation events (>30 mm), which mainly affect deep soil moisture, are account for 48% of the total rainfall during the growing season (Liu et al., 2011). Walter’s two-layer model proposed that water in the deep layers of the soil is ineffective for grasses with shallow roots, and shrubs with deep roots have exclusive access to a source of water in relatively deep layers (Walter, 1979; Walker et al., 1981). Based on this model, we believed that annuals with a more opportunistic strategy, could utilize the short-term availability of water in upper soil layers and rapidly grow to complete their life cycle once the rainfall event occurs in arid and semi-arid areas. Perennial, however, tend to develop a deeper and rigid root system to have access to deeper soil water resources in drier areas as water stress intensifies, and establish smaller leaves to reduce metabolism and leaf gas exchange, as well as to allocate more photosynthate to root build when nutrients are insufficient. With these conservative strategies, perennials have less response to the fluctuation of rainfall in arid and semi-arid areas and are gradually colonized in desertified grassland.

On the vertical direction, vertical resource partitioning interacted with annuals-perennials resource utilization strategy differences, to realize the diversification of soil water resources utilization, which is the driving force behind annuals-perennials coexistence (Holdo, 2013). Furthermore, the colonization of perennial plants in desertified grassland increases resource heterogeneity by forming fertile islands beneath their canopies (Ding and Eldridge, 2021). The perennial patches could act as nurse plants for annuals in arid and semi-arid areas, protecting annuals from windy, dusty, and herbivores (Dohn et al., 2013). On the horizontal direction, spatial differences in resource utilization (e.g., nutrients, moisture, light) resulted in niche partitioning (and therefore coexistence) between annuals and perennials. Therefore, annuals and perennials could coexist stably due to the diversification of their both vertical and horizontal spatial resource utilization in undisturbed habitat, which provides necessary conditions for the colonization of perennials in desertified grassland. However, if the persistently external disturbance is removed, some perennial species may gradually vanish as vegetation succession progressed in the late stage of desertified grassland restoration. For example, the establishment of the protective system of artificial vegetation in Shapotou, China, led to an increase in the percentage of annual herbaceous species, but a decrease in the abundance of others, such as restored (artificial) planted shrubs, as succession progressed. These changes were related to a thickened sand surface crust, the reduced moisture content in the deep layer, and the limited reproduction of deep-rooted shrubs and perennial herbaceous plants (Li et al., 2003).

In the last 40 years, Horqin Sandy Land has been subjected to serious desertification, accompanied by a sharp decline in soil nutrients and marked changes in the vegetation. The dominant species *Cleistogenes squarrosa*, *Chenopodium acuminatum, Tribulus terrester*, *Chloris virgata*, etc., were observed in the early stage of desertification. Then, *Aneurolepidium dasystachys*, *Caragana microphylla*, *Artemisia halodendron*, *Bassia dasyphylla*, *Lespedeza bicolor*, *Pennisetum centrasiaticum* gradually colonized (Ning et al., 2021). This process was closely related to the adaptation and resource utilization strategies of these species. But in the most extreme cases, resulting from continuous disturbances, shrub or thicket (*Artemisia halodendron*) and/or *Agriophyllum squarrosum* dominated the final state, virtually excluding other species (Zhang et al., 2004).

The differences in adaptation, resource acquisition, and allocation strategies between annuals and perennials can also provide guidance for the restoration and management of desertified grassland. In severely desertified grassland, it is necessary to establish artificial sand-fixing vegetation with perennial plants, which have a conservative strategy, such as smaller leaves and a deeper and rigid roots system, and thus can gradually colonize in desertified grassland. The colonization of perennials can create better conditions for the recolonization of annuals, which adopt an opportunistic strategy. Annuals can utilize the short-term availability of water in upper soil layers and rapidly grow to complete their life cycle once the rainfall events occur. Annuals and perennials might show a dynamically balanced state and coexist stably, due to diversification of their both vertical and horizontal spatial resource utilization. Thus, a new artificial-natural ecosystem might be established in the former moving dune-dominated landscape, which is important to the mitigation of desertification and restoration of desertified grassland in arid and semi-arid areas.

## Conclusion

Our study shows important implications for an in-depth understanding of the mechanism of species changes in desertified grassland in arid and semi-arid areas. Annuals and perennials differed considerably in terms of adaptation, resource acquisition, and allocation strategies, which might be partly responsible for species changes in desertified grassland. First, annuals differed from perennials in terms of several leaves and fine root traits important in resource uptake and conservation. Annuals displayed an opportunistic strategy associated with enhanced resource acquisition *via* the presence of large leaves, high SLA and SRL, low-density leaf and fine root, and high N concentration, while perennials exhibited a conservative strategy relatively. Second, annuals tend to establish tough tissue and low palatability, but perennials tend to have smaller leaves to reduce metabolism and water loss in resource-limited conditions. Third, there was a proportional allocation of photosynthate between leaf and fine root in annuals. But perennials showed a conservative resource allocation strategy that species tend to allocate more photosynthate to roots to improve resource absorption capacity in resource-limited habitats, resulting in a deeper and rigid roots system and smaller leaves. Perennials gradually colonized and coexisted with annuals in desertified grassland in arid and semi-arid areas, due to their different conservative adaptation and resource acquisition strategies, as well as their diversified use of spatial resources. These different strategies of annuals and perennials might be useful for the prediction of the succession of vegetation community in desertified grassland in arid and semi-arid areas, and thus provide support for the management and restoration of desertified grassland. We suggest the establishment of artificial sand-fixing vegetation with perennials in desertified grassland, which would facilitate the recolonization of annuals, and is expected to form a stable artificial-natural ecosystem.

## Data Availability Statement

The original contributions presented in the study are included in the article/Supplementary Material, further inquiries can be directed to the corresponding authors.

## Author Contributions

XZ conceived and designed the study based on discussions involving ZN and YL. ZN, YL, DH, and JZ performed the experiments. ZN and YL analyzed the results. ZN drafted the manuscript. All co-authors had a chance to review the manuscript before submission and contributed to discussion and interpretation of the data. All authors contributed to the article and approved the submitted version.

## Conflict of Interest

The authors declare that the research was conducted in the absence of any commercial or financial relationships that could be construed as a potential conflict of interest.

## Publisher’s Note

All claims expressed in this article are solely those of the authors and do not necessarily represent those of their affiliated organizations, or those of the publisher, the editors and the reviewers. Any product that may be evaluated in this article, or claim that may be made by its manufacturer, is not guaranteed or endorsed by the publisher.

## Footnotes

1
https://www.r-project.org/

2
http://camwebb.info/doc/phylomatic.html

## Supplementary Material

Supplementary Table A. 1 The 54 species surveyed in the study in Horqin Sandy Land.

Supplementary Table A. 2 Traits of leaf and fine root for 54 species to different functional groups.

Supplementary Table A. 3 Principal Components Analysis of the 54 species with respect to leaf and fine root traits.
