# Supplementary material for: Comparison of Leaf and Fine Root Traits Between Annuals and Perennials, Implicating the Mechanism of Species Changes in Desertified Grasslands
Source: Front Plant Sci. 2022 Feb 4;12:778547. doi: 10.3389/fpls.2021.778547 (PMC8854787; doi:10.3389/fpls.2021.778547)
Supplement: Supplementary Table A. 1 — The 54 species surveyed in the study in Horqin Sandy Land. [file Table_1.docx]

**Table A. 1** The 54 species surveyed in the study in Horqin Sandy Land

| Species | Life form | Category | Type | Photos-  ynthetic pathway | species | Life form | Category | Type | Photos-  ynthetic pathway |
| --- | --- | --- | --- | --- | --- | --- | --- | --- | --- |
| *Artemisia argyi* | PF | NL | D | C3 | *Ephedra sinica* | SH | NL | - | - |
| *Artemisia annua* | AF | NL | D | C3 | *Erodium stephanianum* | PF | NL | D | C_3_ |
| *Aristida adscensionis* | AG | NL | M | C_4_ | *Hedysarum fruticosum* | SH | L | D | C_3_ |
| *Astragalus adsurgens* | PF | L | D | C_3_ | *Hibiscus trionum* | AF | NL | D | C_3_ |
| *Artemisia frigida* | SH | NL | D | C3 | *Ixeridium gracile* | AF | NL | D | C_3_ |
| *Artemisia halodendron* | SH | NL | D | C_3_ | *Kummerowia striata* | AF | L | D | C_3_ |
| *Allium mongolicum* | PF | NL | M | C_3_ | *Leonurus artemisia* | AF | NL | D | C_3_ |
| *Artemisia sieversiana* | AF | NL | D | C_4_ | *Lespedeza bicolor* | SH | L | D | C_3_ |
| *Agriophyllum squarrosum* | AF | NL | D | C_4_ | *Leymus chinensis* | PG | NL | M | C_3_ |
| *Bassia dasyphylla* | AF | NL | D | C_4_ | *Lespedeza juncea* | SH | L | D | C_4_ |
| *Consolida ajacis* | AF | NL | D | C_3_ | *Leymus secalinus* | PG | NL | M | C_3_ |
| *Chenopodium aristatum* | AF | NL | D | C_4_ | *Medicago ruthenica* | PF | L | D | C_3_ |
| *Chenopodium acuminatum* | AF | NL | D | C_4_ | *Olgaea leucophylla* | PF | NL | D | C_3_ |
| *Cynanchum chinense* | AF | NL | D | C_3_ | *Phragmites australis* | PG | NL | M | C_3_ |
| *Cenchrus echinatus* | AG | NL | M | C_4_ | Pennisetum centrasiaticum | PG | NL | M | C_4_ |
| *Caragana microphylla* | SH | L | D | C_3_ | *Potentilla chinensis* | PF | NL | D | C_4_ |
| *Corispermum macrocarpum* | AF | NL | D | C_3_ | *Portulaca oleracea* | AF | NL | D | C_4_ |
| *Calamagrostis pseudophragmites* | PG | NL | M | C_4_ | *Polygonum tortuosum* | SH | NL | M | C_3_ |
| *Cleistogenes squarrosa* | PG | NL | M | C_4_ | *Salsola collina* | AF | NL | D | C_4_ |
| *Cynanchum thesioides* | AF | NL | D | C_3_ | *Salix gordejevii* | SH | NL | D | C_3_ |
| *Chloris virgata* | AG | NL | M | C_4_ | *Sophora flavescens* | PF | L | D | C_3_ |
| *Digitaria chrysoblephara* | AG | NL | M | C_4_ | *Sonchus oleraceus* | AF | NL | D | C_3_ |
| *Enneapogon borealis* | PG | NL | M | C_4_ | *Setaria viridis* | AG | NL | M | C_4_ |
| *Euphorbia esula* | PF | NL | D | C_4_ | *Thalictrum aquilegifolium* | PF | NL | D | C_3_ |
| *Echinops gmelini* | AF | NL | D | C_3_ | *Tragus racemosus* | AG | NL | M | C_4_ |
| *Euphorbia humifusa* | AF | NL | D | C_4_ | *Tribulus terrester* | PF | NL | D | C_4_ |
| *Eragrostis pilosa* | AG | NL | M | C_4_ | *Xanthium sibiricum* | AF | NL | D | C_4_ |

AG, annual grasses; PG, perennial grasses; AF, annual forbs; PF, perennial forbs; SH, shrub; L, legume; NL, non-legume. D, dicotyledons; M, monocots; C_3_ and C_4_, C_3_ and C_4_ plants.

**Table A. 2** Traits of leaf and fine root for 54 species to different functional groups.

| Trait | AG | PG | AF | PF | SH |
| --- | --- | --- | --- | --- | --- |
| LT (mm) | 0.12±0.01a | 0.14±0.02a | 0.28±0.04a | 0.31±0.11a | 0.33±0.09a |
| LA (cm^2^) | 4.10±0.95a | 9.32±2.42a | 9.25±3.35a | 6.46±3.51a | 1.24±0.50a |
| LDMC (g/g) | 0.26±0.02bc | 0.34±0.02c | 0.17±0.02a | 0.24±0.02b | 0.32±0.04c |
| SLA (cm^2^/g) | 263.44±34.91b | 202.19±24.43ab | 259.08±18.98b | 180.30±17.18a | 156.51±22.93a |
| LTD (g/cm^3^) | 0.04±0.01bc | 0.04±0.01c | 0.02±0.00a | 0.03±0.00b | 0.03±0.01bc |
| LN (%) | 1.64±0.20a | 1.75±0.16a | 2.57±0.12b | 2.68±0.20b | 2.55±0.21b |
| LC (%) | 43.35±0.43ab | 43.82±0.72ab | 42.19±0.94a | 44.28±0.68ab | 46.24±1.17b |
| FRDMC (g/g) | 0.39±0.02b | 0.39±0.03b | 0.29±0.01a | 0.30±0.03a | 0.40±0.03b |
| SRL (cm/g) | 2284.23±316.26c | 1505.20±170.39b | 1194.64±134.95ab | 768.13±96.70a | 1008.31±341.78ab |
| FRTD (g/cm^3^) | 0.14±0.01a | 0.15±0.02a | 0.24±0.01b | 0.28±0.04b | 0.29±0.03b |
| FRN（%） | 0.49±0.11a | 0.66±0.16ab | 0.98±0.07b | 1.42±0.17c | 1.39±0.16c |
| FRC（%） | 45.97±0.42ab | 44.70±0.64a | 45.07±0.34a | 46.15±0.59ab | 47.30±0.56b |

Mean±SE. Different letters indicate significant differences between functional groups (ANOVA followed by LSD test, *P*<0.05). AG, annual grasses; PG, perennial grasses; AF, annual forbs; PF, perennial forbs; SH, shrub. LT, leaf thickness; LA, leaf area; LDMC, leaf dry-matter content; SLA, specific leaf area; LTD, leaf tissue density; FRDMC, fine root dry-matter content; SRL, specific root length; FRTD, fine root tissue density; LN, leaf nitrogen concentration; LC, leaf carbon concentration; FRN, fine root nitrogen concentration; FRC, fine root carbon concentration.

**Table A. 3** Principal Components Analysis of the 54 species with respect to leaf and fine root traits

| PCA without PIC | | | | | PCA with PIC | | | |
| --- | --- | --- | --- | --- | --- | --- | --- | --- |
| Variables | Axis1  EV(0.275) | Axis2  EV(0.214) | Axis3  EV(0.113) | Axis4  EV(0.105) | Axis1  EV(0.364) | Axis2  EV(0.206) | Axis3  EV(0.130) | Axis4  EV(0.092) |
| LT | **-0.683** |  |  | 0.510 |  | **-0.949** |  |  |
| LA |  |  | **0.837** |  | **0.462** | 0.291 |  | 0.445 |
| LDMC | **0.844** |  |  | 0.316 | 0.477 | **0.696** | 0.316 | -0.348 |
| SLA |  |  |  | **-0.925** |  |  | **-0.944** |  |
| LTD | **0.897** |  |  | 0.260 | -0.258 | 0.350 | **0.823** |  |
| LN |  | **0.861** |  |  | **0.816** |  |  | 0.281 |
| LC | **0.645** | 0.416 |  |  |  | **0.929** |  |  |
| FRDMC | **0.645** |  | 0.391 |  | -0.379 |  |  | **-0.609** |
| SRL |  | **-0.590** | 0.474 |  |  |  |  | **0.773** |
| FRTD |  | 0.474 | **0.497** | 0.286 | **-0.711** |  | 0.278 | -0.319 |
| FRN |  | **0.794** |  |  | **0.907** |  |  |  |
| FRC | 0.415 | 0.266 | **0.460** |  | **-0.867** |  |  |  |

Values are the loadings of the selected variables along the fore most important explanatory axes. Numbers in bold marked the variables with the closest correlation to the respective axis. LT, leaf thickness; LA, leaf area; LDMC, leaf dry-matter content; SLA, specific leaf area; LTD, leaf tissue density; FRDMC, fine root dry-matter content; SRL, specific root length; FRTD, fine root tissue density; LN, leaf nitrogen concentration; LC, leaf carbon concentration; FRN, fine root nitrogen concentration; FRC, fine root carbon concentration.
